# The interaction of genetic determinants in the outcome of HCV infection: evidence for discrete immunological pathways

**DOI:** 10.1111/tan.12650

**Published:** 2015-09-18

**Authors:** T. J. Hydes, B. Moesker, J. A. Traherne, S. Ashraf, G. J. Alexander, B. D. Dimitrov, C. H. Woelk, J. Trowsdale, S. I. Khakoo

**Affiliations:** ^1^ Clinical & Experimental Sciences, Faculty of MedicineUniversity of SouthamptonSouthamptonUK; ^2^ Department of PathologyUniversity of CambridgeCambridgeUK; ^3^ Department of MedicineUniversity of CambridgeCambridgeUK; ^4^ Academic Unit of Primary Care and Population Sciences, Faculty of MedicineUniversity of SouthamptonSouthamptonUK

**Keywords:** hepatitis C, IFN‐λ3/4, immunogenetics, interferon, killer cell immunoglobulin receptors, tapasin

## Abstract

Diversity within the innate and adaptive immune response to hepatitis C is important in determining spontaneous resolution (SR) and treatment response. The aim of this study was to analyze how these variables interact in combination; furthering our understanding of the mechanisms that drive successful immunological clearance. Multivariate analysis was performed on retrospectively collected data for 357 patients previously genotyped for interferon (IFN)‐λ3/4, killer cell immunoglobulin (KIR), human leukocyte antigen (HLA) class I and II and tapasin. High resolution KIR genotyping was performed for individuals with chronic infection and haplotypes determined. Outcomes for SR, IFN response and cirrhosis were examined. Statistical analysis included univariate methods, χ^2^ test for trend, multivariate logistic regression, synergy and principal component analysis (PCA). Although KIR2DL3:HLA‐C1C1 (P = 0.027), IFN‐λ3/4 rs12979860 CC (P = 0.027), tapasin G in individuals with aspartate at residue 114 of HLA‐B (TapG:HLA‐B^114D^) (P = 0.007) and HLA‐DRB1*04:01 (P = 0.014) were associated with SR with a strong additive influence (χ^2^ test for trend P < 0.0001); favorable polymorphisms did not interact synergistically, nor did patients cluster by outcome. In the treatment cohort, IFN‐λ3/4 rs12979860 CC was protective in hepatitis C virus (HCV) G1 infection and KIR2DL3:HLA‐C1 in HCV G2/3. In common with SR, variables did not interact synergistically. Polymorphisms predictive of viral clearance did not predict disease progression. In summary, different individuals resolve HCV infection using discrete and non‐interacting immunological pathways. These pathways are influenced by viral genotype. This work provides novel insights into the complexity of the interaction between host and viral factors in determining the outcome of HCV infection.

## Introduction

Only 20% of individuals exposed to hepatitis C virus (HCV) achieve spontaneous resolution (SR) and despite the recent revolution in new drug therapies, HCV remains a common cause of cirrhosis and liver cancer worldwide with no effective vaccine in immediate sight 1, 2. Genetic and functional studies have identified key molecules and processes involved in clearing HCV spontaneously and using interferon (IFN)‐α based therapies 3, 4, 5, 6, 7, 8, 9. These studies have implicated both innate and adaptive mechanisms in clearing HCV infection.

One of the most impressive findings from Gene Wide Association Studies (GWAS) to date has been the identification of single nuclear polymorphisms (SNPs) upstream of IFN‐λ3 which are protective against HCV chronicity and treatment failure 3, 4, 5, 6, 7. This has been fine mapped to the TT/‐G polymorphism at rs67272382 in a CpG island, which is in strong linkage disequilibrium with IFN‐λ3 and is most likely to be the causal variant, with the possibility of defining a new type 3 IFN, IFN‐λ4 10, 11. Whilst this polymorphism has shown remarkable consistency in its ability to predict spontaneous and treatment‐induced viral clearance across populations, its protection does not extend to ‘exposed uninfected’ individuals, suggesting that multiple pathways to clearance may coexist within a population 12, 13.

The killer cell immunoglobulin (KIR) and human leukocyte antigen (HLA) combinations are also important for HCV outcomes in spontaneously resolving infection, IFN‐treatment‐associated resolution and in European‐exposed seronegative individuals 8, 14. In these studies, the combination of KIR2DL3 and its HLA‐C group 1 ligands (HLA‐C1) was protective in all groups tested in our UK population. These findings have been confirmed in sub‐groups in other populations, but it is clear that there is population diversity in how the KIR gene family contributes to the resolution of HCV infection 15. The influence of KIR diversity remains important in the era of triple therapy, until directly acting anti‐viral therapies become cost effective 16, 17.

Within our Caucasian population we recently identified that an SNP in tapasin, a protein involved in the major histocompatibility complex (MHC) class I peptide loading pathway was associated with resolution of HCV infection 9. The favorable tapasin G gene was most protective in individuals carrying HLA‐B alleles with an aspartate at residue 114 of the HLA‐B heavy chain (TapG:HLA‐B^114D^). Individuals with a protective allele had a stronger T‐cell response. Thus, both the adaptive and innate immune systems are protective against chronic HCV infection in our UK population. This is borne out by other studies indicating a role for HLA‐class II polymorphisms in the outcome of HCV infection in the UK 18, 19.

Recent work has studied how the innate immune pathways of KIR, HLA‐C and IFN‐λ3/4 interact. In these studies it has been shown that for the SNPs associated with worse outcomes, KIR2DS3, HLA‐C2C2 and IFN‐λ3/4 (rs8099917 G allele and rs12979860 T allele), there appeared to be an interaction, as individuals with two susceptibility factors had significantly worse outcomes than those with only one 20, 21. Conversely no predictive benefit was found on combining the favorable IFN‐λ3/4 polymorphism with KIR2DL3:HLA‐C1 homozygosity for protection against viremia in seronegative exposed groups 12. On examination of the interaction of class I and II HLA alleles with KIR and IFN‐λ3/4 in the homogenous Irish HCV cohort and more heterogenous Swiss cohort, nothing further than an additive effect was seen 22.

Viral genotype is emerging as a central determinant of whether the above polymorphisms can exert any influence on viral clearance; for example variants of IFN‐λ3/4 play a limited role in predicting HCV related outcomes in patients infected with HCV G2/3 23. Recent work has suggested this may be a result of genotype 1 viral infection inducing transcripts which are heavily regulated by IFN‐λ3/4, whereas HCV G2/3 infection leads to the upregulation of genes induced by IFN‐γ 24. There was no influence of IFN‐λ3/4 polymorphisms on the transcriptional profile of patients infected with HCV G3 24. Viral genotype specific host‐pathogen interactions are therefore vital to consider when studying the impact of host immunogenetics in HCV.

Thus clearance of hepatitis C depends on both innate and adaptive immune systems implicated in both genetic and cellular studies. However it has been difficult to study how these systems interact. To do this we have studied our UK HCV  Caucasian population, which has been extensively typed for polymorphic gene families KIR, HLA‐I, HLA‐II and IFN‐λ3/4.

## Materials and methods

A total of 357 patients were included in the study recruited from two large UK teaching hospitals, University Hospitals Southampton NHS Foundation Trust and Cambridge University Hospitals NHS Foundation Trust between 2003 and 2007 with ethical approval. Patients were recruited from hepatology clinics having tested positive for HCV IgG by second generation enzyme‐linked immunosorbent assay (ELISA). Viremia was confirmed using the HCV Cobas Amplification system (Roche). SR was defined as the absence of HCV RNA, 6 months following a positive antibody test. HCV genotyping was performed using quantitative polymerase chain reaction (PCR) (iQur, Southampton, UK) 9, 12, 14.

DNA was isolated as previously described. Genotyping had previously been performed for KIR, IFN‐λ3/4 rs12979860, HLA and tapasin 9, 12, 14. Additional high resolution KIR genotyping was later performed on the patients with chronic HCV infection to accurately determine KIR copy number and impute KIR haplotype 25. Demographic data and primary end points [spontaneous and treatment‐induced viral clearance, the development of cirrhosis and hepatocellular carcinoma (HCC)] were collected retrospectively by the researchers using the patient's electronic medical records.

Statistical analysis of the numeric variables was performed using descriptive statistics [mean, standard deviation, 95% confidence intervals (CI)] while that for the categorical variables (frequency, percent) was performed using frequency distribution and cross‐tabulation methods, as appropriate. Univariate and multivariate analyses were further performed to determine factor selection and test independence. The multivariate logistic regression was applied using the backward stepwise procedure for all variables that were significant at univariate analysis. The relationships were estimated using the odds ratio (OR) and its 95% CI. Chi‐squared test for trend and Fischer's exact test were also used to analyze the benefit of having more than two protective variables. The interaction between susceptibility or protective factors was reported using both the synergy factor (SF), as described by Cortina‐Borja et al. 26 and the synergy index (SI) 27. A *P* < 0.05 was considered to be statistically significant, unless stated otherwise. Routine statistical software packages were used for the analyses (SPSS version 21, GraphPad Prism). Three‐dimensional PCA (*rgl* package in R) was used to cluster patients on the basis of their SNP profiles 28, 29.

## Results

### Spontaneous resolution

We had previously typed our HCV population for SNPs affecting both innate and adaptive pathways that are associated with protection or susceptibility to HCV infection. These include HLA‐I, HLA‐II, KIR, tapasin and IFN‐λ3/4 as described previously 9, 12, 14. Broadly speaking these represent a cellular innate immune response (IFN‐λ3/4), cytotoxic T‐lymphocytic response (HLA‐I and tapasin), T helper responses (HLA‐II) and NK cells (KIR), all of which have been implicated in the outcome of HCV infection. Our cohort to study SR included 61 resolvers and 296 chronic individuals. Genetic polymorphisms reaching significance in univariate analysis (Table S1, Supporting Information) were entered into a multivariate binary logistic regression model. The following variables remained independently significant: KIR2DL3:HLA‐C1C1 (*P* = 0.006, OR 3.05, 95% CI 1.38–6.74), IFN‐λ3/4 rs12979860 CC (*P* = 0.013, OR 2.55, 95% CI 1.22–5.31), HLA‐DRB1*04:01 (*P* = 0.011, OR 3.70, 95% CI 1.36–10.08) and TapG:HLA‐B^114D^ (*P* = 0.008, OR 3.22, 95% CI 1.36–7.65) and KIR2DS5 was susceptible (*P* = 0.027, OR 0.37, 95% CI 0.16–0.90) (Table 1).

**Table 1:** Spontaneous resolution (individual polymorphisms)

				Multivariate logistic regression	
Genetic factor	Genotype	Resolved *n* = 61	Chronic *n* = 296	*P*‐value	OR (95% CI)
IFN‐λ3/4 rs12979860	CC	34	116	0.013	2.55 (1.22–5.31)
KIR:HLA	2DL3:HLA‐C1C1	24	70	0.006	3.05 (1.38–6.74)
	2DS3	7	71	0.114	0.45 (0.17–1.21)
	2DS5	11	87	0.027	0.37 (0.16–0.90)
	HLA‐DRB1*04:01	14	12	0.011	3.70 (1.36–10.08)
Tapasin & HLA	TapG:HLA‐B^114D^	48	148	0.008	3.22 (1.36–7.65)

CI, confidence intervals; HLA, human leukocyte antigen; KIR, killer cell immunoglobulin; OR, odds ratio.

**Figure 1:**
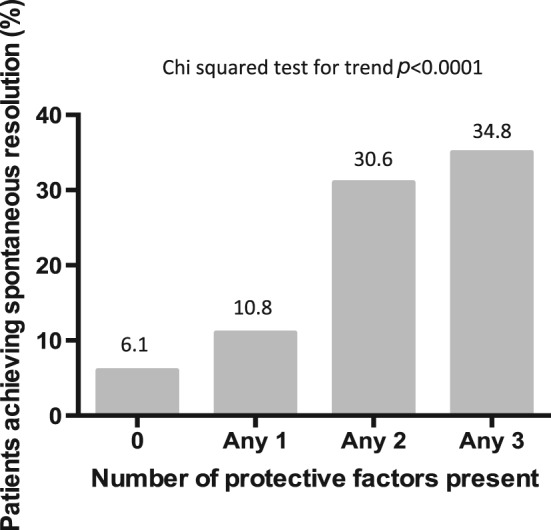
The combination of IFN‐λ3/4 rs12979860 CC, KIR2DL3:HLA‐C1 and TapG:HLA‐B^114D^ in determining spontaneous viral clearance.

We next examined how these genes interacted in combination to bring about clearance of HCV. Overall, 6.1% (2/33) of individuals with no protective factors, 10.8% (12/111) of individuals with one protective factor, 30.6% (30/98) with two protective factors and 34.8% (8/23) with three protective factors resolved infection, *P* < 0.0001 chi‐squared for trend (Figure 1). To study whether this additional protection was because of the random association of protective factors acting independently or a synergistic effect of these genes working to augment a single immunological pathway, we calculated *P*‐values and ORs based on the occurrence of the combination of protective factors in the population, and performed two types of synergy analysis, the SI and SF (Table 2) 26, 27. Both synergy tools are designed to account for the expected increase in protection due to the chance occurrence of the combination of factors. A SI > 1 indicates an interaction of either two negative or two positive factors, but a SI of 1 shows no interaction. Conversely for the SF, synergism of protective factors produces a SF < 1 and synergism of disadvantageous factors produces a SF > 1. In both analyses, no statistically significant synergistic interactions were found, and only a weak trend towards TapG:HLA‐B^114D^ and IFN‐λ3/4 rs12979860 CC interacting synergistically for SR (SF 0.36, *P* = 0.085, 95% CI 0.11–1.15; SI 6.95, 95% CI 0.06–752.17) was found. All other combinations gave null results, with SF > 1 and SI < 1, including the combination of HLA‐DRB1*04:01 with TapG:HLA‐B^114D^ and IFN‐λ3/4 rs12979860 C despite together significantly strengthening their OR for bringing about resolution, unlike the pairing of KIR2DL3:HLA‐C1C1 with TapG:HLA‐B^114D^ or IFN‐λ3/4 rs12979860 CC (Tables 1 and 2). Thus for protection against chronic HCV, we propose that these factors are acting independently in resolving HCV infection using discrete immunological pathways.

**Table 2:** Spontaneous resolution (polymorphisms combined)

	Univariate analysis		Synergy factor			Synergy index	
Genotype	*P*‐value	OR (95% CI)	Synergy factor	*P*‐value	95% CI	Synergy index	95% CI
TapG:HLA‐B^114D^ + IFN‐λ3/4 rs12979860 CC	<0.001	3.84 (2.10–7.03)	0.36	0.085	0.11–1.15	6.95	0.06–752.17
TapG:HLA‐B^114D^ + KIR2DL3:HLA‐C1C1	0.012	2.37 (1.21–4.67)	2.83	0.194	0.59–13.56	0.97	0.41–2.32
IFN‐λ3/4 rs12979860 CC + KIR2DL3:HLA‐C1C1	0.070	1.98 (0.95–4.13)	3.32	0.067	0.92–11.95	0.59	0.20–1.70
IFN‐λ3/4 rs12979860 CC + HLA‐DRB1*04:01	0.004	5.10 (1.69–15.35)	1.92	0.473	0.32–11.44	0.95	0.17–5.30
TapG:HLA‐B^114D^ + HLA‐DRB1*04:01	0.001	4.51 (1.79–11.36)	1.81	0.612	0.18–17.82	1.00	0.12–8.06
KIR2DL3:HLA‐C1C1 + HLA‐DRB1*04:01	0.999	6.2 × 10^9^ (0‐.)	N/A	N/A	N/A	N/A	N/A
KIR2DS3 + KIR2DS5	0.240	0.48 (0.14–1.64)	0.29	0.1698	0.049–1.70	0.44	0.06–3.37

CI, confidence intervals; OR, odds ratio.

### Sustained virological response to pegylated IFN and ribavirin

To determine if immunological pathways acted discretely or in concert in treatment‐induced responses, and also to determine the effect of viral genotype, we performed multivariate backward stepwise logistic regression (Table 3) on factors reaching significance in univariate analysis (Table S2) in patients who had undergone treatment with available sustained virological response (SVR) outcome data. To gain additional information on KIR in our population, we typed 185 patients who had undergone treatment with pegylated IFN and ribavirin with a high resolution KIR typing method (Table S3). This allowed both gene copy number and haplotype assignment to the cohort 25. Briefly KIR haplotypes can be categorized as centromeric and telomeric A and B haplotypes according to the classification of Cooley et al. 30 As individual KIR genes are in strong linkage disequilibrium with each other, these haplotypes may be more strongly associated with disease outcome than individual genes. We had complete data on 91 patients with HCV genotype 1 infection and 94 with HCV genotype 2 or 3 infection who had undergone treatment with pegylated IFN/ribavirin dual therapy. As response to IFN is HCV genotype dependent, we considered the cohort both as a whole and also divided into those with genotype 1 *vs* those with genotype 2/3 infection.

**Table 3:** Sustained virological response to pegylated interferon/ribavirin (individual polymorphisms)

				Multivariate logistic regression	
	Genotype	SVR *n* = 105	No SVR *n* = 80	*P*‐value	OR (95% CI)
All genotypes	IFN‐λ3/4 rs12979860 CC	42	23	0.265	1.50 (0.73–3.08)
	KIR3DS1:HLA‐Bw4^801^	14	3	0.016	5.23 (1.35–20.19)
	KIR2DL2/S2:HLA‐C1C1	11	19	0.018	0.30 (0.11–0.82)
	KIR2DL2	45	47	0.742	0.88 (0.41–1.90)
HCV G1	IFN‐λ3/4 rs12979860 CC	15	11	0.043	3.11 (1.04–9.32)
	CA01CA01	17	15	0.098	2.51 (0.84–7.49)
HCV G2/3	KIR2DL3:HLA‐C1	50	15	0.018	4.90 (1.32–18.25)
	CB01	9	9	0.137	0.38 (0.11–1.36)

CI, confidence intervals; OR, odds ratio; SVR, sustained virological response.

After multivariate logistic regression analysis only KIR3DS1:HLA‐Bw4^80I^ (*P* = 0.016, OR 5.23, 95% CI 1.35–20.19) and KIR2DL2/S2:HLA‐C1C1 (*P* = 0.018, OR 0.30, 95% CI 0.11–0.82) were identified as independent predictors of outcome in the whole cohort, the first being protective and the latter leading to treatment failure. In addition, IFN‐λ3/4 rs12979860 CC (*P* = 0.043, OR 3.11, 95% CI 1.04–9.32) and KIR2DL3:HLA‐C1 (*P* = 0.018, OR 4.90, 95% CI 1.32–18.25) were protective in HCV in genotype 1 infection and HCV genotype 2 or 3 infection, respectively (Table 3). Despite not reaching independent significance, homozygosity for centromeric motifs of KIR haplotype A (CA01), which contains KIR2DL3 (*P* = 0.041, OR 2.74, 95% CI 1.04–7.20) were associated with treatment response in univariate analysis in HCV G1 infection and the CB01 haplotype (which contains KIR2DL2) was closely associated with an absence of SVR in G2/3 infection (*P* = 0.051, OR 0.33, 95% CI 0.11–1.01) (Table S2). We observed no association with any polymorphisms in HLA‐I or HLA‐II or tapasin, indicating that innate immune response are dominant in IFN‐related clearance, but that G1 HCV and G2/3 HCV use discrete innate pathways.

Consistent with our data in spontaneous resolvers, the combination of protective factors generated from univariate analysis for all HCV genotypes, IFN‐λ3/4 rs12979860 CC and KIR3DS1:HLA‐Bw4^80I^, did not result in additional benefit (SF 2.60, 95% CI 0.17–39.02; SI 0.39, 95% CI 0.02–7.44, respectively) (Table 4). As a result of susceptibility factors, KIR2DL2 and KIR2DS2 being in linkage disequilibrium at neighboring loci, with all patients with KIR2DL2 also carrying KIR2DS2, synergy calculation was precluded for these variables. The absence of synergy is also present within the HCV genotype 1 cohort, with no significant synergistic interaction being found between homozygosity for centromeric components of KIR haplotype A and IFN‐λ3/4 rs12979860 CC (SF 0.39, 95% CI 0.04–4.20; SI 7.80, 95% CI 0.24–256.69). Therefore in common with SR, discrete genetic pathways operate to bring about IFN induced HCV clearance within an individual. While these pathways appear to act independently of each other their influence is strongly linked to HCV genotype.

**Table 4:** Sustained virological response to pegylated interferon/ribavirin (polymorphisms combined)

	Univariate analysis			Synergy factor			Synergy index	
Genotype	Polymorphism combination	*P*‐value	OR (95% CI)	Synergy factor	*P*‐value	95% CI	Synergy index	95% CI
All genotypes	IFN‐λ3/4 rs12979860 CC + KIR3DS1:HLA‐Bw4^801^	0.181	2.98 (0.60–14.75)	2.60	0.490	0.17–39.02	0.39	0.02–7.44
	KIR2DL2/S2:HLA‐C1C1 + KIR2DL2	0.014	0.36 (0.16–0.81)	All patients with KIR2DL2 have KIR 2DS2			All patients with KIR2DL2 have KIR 2DS2	
HCV G1	IFN‐λ3/4 rs12979860 CC + CA01CA01	0.009	6.55 (1.61–26.56)	0.39	0.435	0.04–4.20	7.80	0.24–256.69

CI, confidence intervals; OR, odds ratio.

To ask the question whether there was an association of HCV genotype with any of these protective factors, we compared the gene frequencies of these polymorphisms within the G1 and G2/3 groups. We found that KIR2DS1, KIR2DS5 and KIR3DS1 (all components of KIR B telomeric haplotypes) were relatively over‐represented in the G2/3 group suggesting that these polymorphisms may lead to susceptibility to chronic infection with G2/3 HCV (Table 5). The correlation between fixed telomeric haplotype B KIR alleles and HCVG2/3 may be explained by the association of motifs of haplotype B with failure to reach both spontaneous and treatment‐induced clearance, leading to a population bias, despite analysis of B haplotypes directly not being significantly associated with HCV genotype; CB01 *P* = 0.365 (OR 0.74, 95% CI 0.39–1.41) and TB01 *P* = 0.098 (OR 1.64, 95% CI 0.91–2.95).

**Table 5:** Genetic polymorphisms found to be differentially represented between HCV G1 and HCV G2/3 infected patients

	HCV G1 Actual count (*expected* * count*)	HCV G23 Actual count (*expected* * count*)	Univariate analysis (*P*‐value)	OR (95% CI)
KIR2DS1	36 (*43.9*)	55 (*47.1*)	0.033	1.80 (1.05–3.10)
KIR2DS1:C2	17 (*24.6*)	33 (*26.2*)	0.022	2.16 (1.12–4.18)
KIR2DS5	25 (*33.4*)	44 (*36.1*)	0.020	2.00 (1.12–3.58)
KIR3DS1	33 (*40.6*)	51 (*43.6*)	0.039	1.78 (1.03–3.09)

CI, confidence intervals; OR, odds ratio.

### Principal component analysis

**Figure 2:**
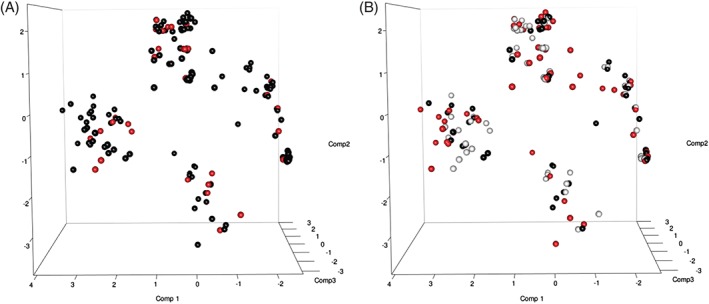
Overlay of hepatitis C outcome data on principal component analysis plots. (A) Spontaneous resolution. Red circles: spontaneous resolution; black circles: chronic infection. (B) Treatment‐induced resolution. Red circles: sustained virological response; black circles: no sustained virological response; gray circles: lost to follow‐up or not treated.

**Figure 3:**
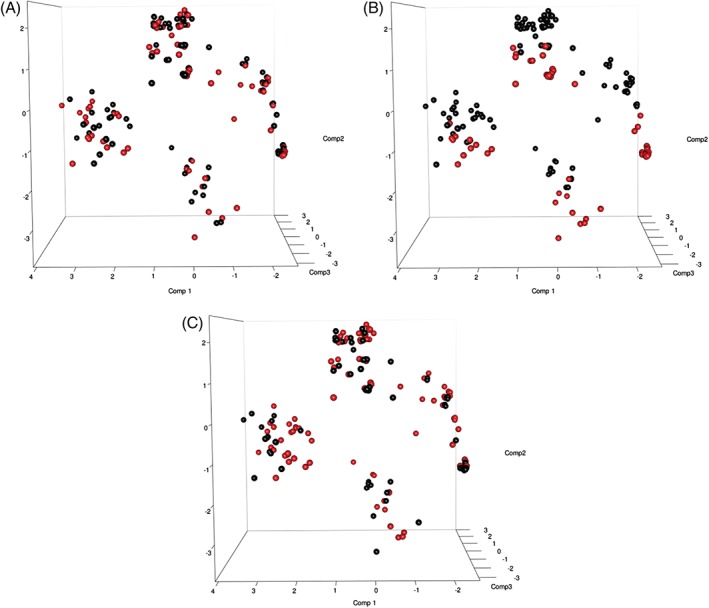
Overlay of single nuclear polymorphisms known to be important for hepatitis C clearance on principal component analysis plots. (A) IFN‐λ3/4 rs12979860 CC, (B) KIR2DL3:HLAC1C1 and (C) TapG:HLA‐B^114D^. Red circles: polymorphism present; black circles: polymorphism absent.

To test conclusions from our synergy analysis, we assessed whether patients with similar binary outcomes for KIR, IFN‐λ3/4, HLA alleles and the tapasin G/C polymorphism would cluster according to outcome using PCA. Prior to PCA analyses SNPs were removed that were not measured for the majority of patients to exclude patients with missing values. In addition, KIRs with multiple copy numbers were excluded to retain the binary data required for PCA, leaving a total of 192 patients and 102 SNPs. While individuals could be seen to cluster into four groups, overlaying outcome data for both SR and SVR could not explain this clustering, providing additional evidence of an absence of anything further than an additive benefit between SNPs from different pathways to resolution (Figure 2). Patients also did not cluster according to previously identified SNPs thought to be key to resolution including IFN‐λ3/4 rs12979860 CC, KIR2DL3:C1C1 and TapG:HLA‐B^114D^ (Figure 3) suggesting that presence of one isolated polymorphism is not enough to significantly define the rest of a patient's genome. Clusters were only found to be linked to KIR haplotypes and some of their defining KIR alleles, KIR2DL2, KIR2DS3, KIR2DS5 and KIR3DS1:HLA‐Bw4^80I^.

### Disease progression

In order to investigate whether similar polymorphisms were associated with disease progression, we analyzed the frequency of IFN‐λ3/4 rs12979860 CC, TapG:HLA‐B^114D^, KIR and HLA polymorphisms known to be important for HCV resolution in patients with chronic hepatitis C who developed cirrhosis compared with those who did not. An outcome was available for 286 patients, of which 78 were cirrhotic. Cirrhosis was defined histologically in 56 patients (71.8%). In those who did not have a biopsy, cirrhosis was defined using a fibroscan score of greater than 12 kPa in three patients (3.8%) and an AST to platelet ratio index (APRI) score of greater than one in the remaining 19 (24.4%). Investigating all the SNPs associated with resolution of HCV using univariate analysis, we found only the centromeric KIR haplotype B to be associated with cirrhosis free survival (*P* = 0.034, OR 2.25, 95% CI 1.06–4.77) (Table 6). On multivariate analysis of significant polymorphisms, the inhibitory KIR2DL2 allele, a component of centromeric KIR B haplotypes, was protective (*P* = 0.029, OR 2.94, 95% CI 1.12–7.73). Thus, factors determining disease progression are distinct from those determining both spontaneous and treatment‐induced resolution. Interestingly of the eight cirrhotic patients who developed HCC and had KIR genotyping available, none carried a centromeric B haplotype, but this was not statistically significant (*P* > 0.1).

**Table 6:** Genetic polymorphisms in disease progression

			Univariate analysis		Multivariate logistic regression	
Genotype	Cirrhosis *n* = 78	No cirrhosis *n* = 208	*P*‐value	OR (95% CI)	*P*‐value	OR (95% CI)
IFN‐λ3/4 rs12979860 CC	36	74	0.174	0.69 (0.40–1.18)	0.776	0.86 (0.30–2.48)
TapG:HLA‐B^114D^	39	102	0.597	1.17 (0.66–2.07)	0.637	1.29 (0.45–3.69)
KIR2DL3:HLA‐C1	45	131	0.393	1.34 (0.69–2.61)	0.831	0.85 (0.19–3.77)
KIR2DL3:HLA‐C1C1	23	47	0.315	0.73 (0.40–1.34)	0.399	0.63 (0.21–1.85)
KIR3DS1:HLA‐Bw4^801^	7	18	0.884	0.93 (0.37–2.34)	0.336	0.44 (0.08–2.36)
KIR2DL2	32	114	0.071	1.65 (0.96–2.86)	0.029	2.94 (1.12–7.73)
KIR2DS2:HLA‐C1C1	11	31	0.695	1.16 (0.55–2.46)	0.772	0.76 (0.12–4.85)
KIR2DS3	15	54	0.106	1.71 (0.89–3.29)	0.928	1.13 (0.88–14.45)
KIR2DS5	25	60	0.981	0.99 (0.56–1.77)	0.332	1.72 (0.58–5.15)
CA01CA01	33	72	0.108	0.62 (0.34–1.11)	0.792	1.48 (0.81–27.0)
CB01	10	53	0.034	2.25 (1.06–4.77)	0.553	1.61 (0.33–7.76)
HLA‐DRB1*04:01	2	10	0.401	1.96 (0.41–9.47)	0.182	4.39 (0.50–38.62)

CI, confidence intervals; OR, odds ratio.

## Discussion

We have studied a well characterized homogenous UK population, in which a number of genetic determinants known to influence the outcome of HCV infection have been previously identified. On a worldwide basis, different populations have different genetic factors that influence outcome of HCV infection. This cohort has provided us with an opportunity to investigate how different protective factors interact in a single cohort at an immunological level. We have studied different outcomes of HCV infection using different statistical tools in order to ensure that our primary finding, that these pathways do not appear to interact, is robust.

Although patients achieve spontaneous clearance through IFN‐λ3/4, TapG:HLA‐B^114D^, KIR2DL3:HLA‐C1 or HLA‐class II (HLA‐DRB1*04:01), these pathways operate independently. Thus, although having more than one protective SNP appears beneficial statistically (Figure 1), these interactions are additive, i.e. because of the random occurrence of two protective genes, rather than synergistic, in which the interaction of two or more genes would produce a combined effect greater than the sum of their separate effects on HCV resolution. We used stringent criteria to define protective interactions, including an improvement in the OR of resolving HCV with the tested gene combinations, two types of synergy analysis and PCA, which is designed to assess the covariance structure of a data set composed of binary outcomes. Overall, none of the individually protective or susceptible genes met these criteria in combination. These data imply that the genetic pathways that lead to resolution of HCV infection are multiple and relatively discrete. Previous work has shown that combining KIR centromeric A/A genotypes with IFN‐λ3/4 improves its positive predictive value in determining treatment outcome 31. However, this is at the expense of a lower sensitivity, and the authors did not test whether this interaction was synergistic or not. Conversely, susceptibility factors appear to increase susceptibility. This was found in an Irish cohort, but not a larger international study 20, 21. Similarly, considering viral genotype and the response to IFN‐based therapy, IFN‐λ3/4 and centromeric A KIR haplotypes are important for treatment‐induced resolution of patients infected with HCV G1, whereas the KIR2DL3 and HLA‐class I combination, but not IFN‐λ3/4, plays a dominant role in resolving HCV G2 and G3 infection. Thus, subtly different pathways operate for different viral genotypes.

While our data infer that pathways to resolution and non‐resolution act independently of each other, it is also likely that diversity within the HCV host immune response seen between discrete populations has a significant effect. Heterogeneity at a population level has continued to contribute to substantial variation seen between studies in HCV resolution. For example for KIR in the UK population, KIR2DL3:HLA‐C1 is protective 8, but this is not found in the cohort of Irish women infected from a single source 20. In a Swiss cohort, the rs8099917 IFN‐λ3/4 minor allele failed to show any association with treatment in patients infected with HCV G2/3, but this was not the case when later repeated in a cohort of over 1000 Australians, also of Caucasian ethnicity 6, 32. In terms of HLA‐class I, HLA‐B*08 has been linked to viral persistence in the Irish HCV and an US cohort, but could not be replicated in an East German population 33, 34, 35. HLA‐DQB1*0301 was found to be protective in largely Caucasian cohorts in France, the UK and Italy, but only in black populations in the United States, showing that geographical location, and hence the infecting HCV virion may be a determinant of response rather than ethnicity alone 18, 36, 37, 38. Finally, the protective influence of tapasin occurs in European Caucasians but not US Caucasians 9.

Our study has shown an important role of KIR haplotypes in HCV resolution, with the assay used being able to assign KIR haplotypes as opposed to simply gene content. These diverse haplotypes have been broadly split into A and B groupings, with the former having one or no functional activating KIR and the latter having several activating KIR. The B group of haplotypes contains genes associated with chronic HCV infection and poor treatment response, including KIR2DL2 and KIR2DS2, but has a minor protective role against end stage manifestations including cirrhosis and HCC, possibly through the activation of NK cells leading to stellate cell apoptosis in the case of fibrosis 39, 40, 41, 42. Activating KIR, components of the KIR B haplotypes, have been previously shown to have a beneficial effect against HCC development 42. We found no other association between genes which influence viral clearance and disease progression.

This work advances our knowledge of how immune pathways, influenced by key genetic polymorphisms, act independently of each other to bring about both spontaneous and treatment‐induced viral clearance in a single Caucasian population. We also show that population diversity within the immune response is likely to account for discordant results between studies, and that while consideration of large populations may be important for discovering widespread protective factors, the study of discrete populations is likely to be important for defining additional protective alleles. Future designers of HCV immunogenetic studies will need to consider stratifying patients according to major characteristics of populations including ethnicity, and be aware that findings are likely to be population discrete. Our results highlight the effect of HCV genotype on dictating whether recognized protective SNPs will be able to influence viral clearance as an interesting topic for further investigation. From a clinical perspective, our data implies the utilization of immunogenetics to guide IFN‐based therapy will require knowledge of the HCV genotype and a personalized approach and that there is no ‘super’ combination of SNPs that can be used to strongly enhance our current predictive models.

## Conflict of interest

The authors have declared no conflicting interests.

## Supporting information


**Table S1.** Univariate analysis of single nuclear polymorphisms leading to spontaneous resolution.

**Table S2.** Univariate analysis of single nuclear polymorphisms leading to a sustained virological response.

**Table S3.** Genetic data available for high resolution KIR typing and treatment related outcomes presented according to haplotype (n = 185).
