# Supplementary material for: The interaction of genetic determinants in the outcome of HCV infection: evidence for discrete immunological pathways
Source: Tissue Antigens. 2015 Sep 18;86(4):267–75. doi: 10.1111/tan.12650 (PMC4858811; doi:10.1111/tan.12650)
Supplement: Supplementary file 1 — Table S1. Univariate analysis of single nuclear polymorphisms leading to spontaneous resolution. [file TAN-86-267-s001.pdf]

Table 1. Univariate analysis of single nuclear polymorphisms leading to spontaneous resolution

Genetic Factor	Genotype	Univariate logistic regression	
		**P-value**	**OR (95% CI)**
IFN-λ3/4 rs12979860	CC	0.027	1.92 (1.08-3.44)
KIR:HLA	2DL3:HLA-C1C1<br>2DS3<br>2DS5<br>HLA-DRB1*04:01	0.027<br>0.015<br>0.026<br>0.014	1.96 (1.08-3.56)<br>0.36 (0.15-0.82)<br>0.45 (0.22-0.91)<br>2.88 (1.24-6.70)
Tapasin & HLA	TapG:HLA-B^{114D}	0.007	2.57 (1.30-5.08)
