# Supplementary material for: The interaction of genetic determinants in the outcome of HCV infection: evidence for discrete immunological pathways
Source: Tissue Antigens. 2015 Sep 18;86(4):267–75. doi: 10.1111/tan.12650 (PMC4858811; doi:10.1111/tan.12650)
Supplement: Supplementary file 2 — Table S2. Univariate analysis of single nuclear polymorphisms leading to a sustained virological response. [file TAN-86-267-s002.pdf]

Table 2. Univariate analysis of single nuclear polymorphisms leading to a sustained virological response

	Genotype	Univariate analysis	
		P-value	OR (95% CI)
All Genotypes	IFN-λ3/4 rs12979860 CC<br>KIR3DS1:HLA-Bw4[801]<br>KIR2DL2/S2:HLA-C1C1<br>KIR2DL2	0.044<br>0.029<br>0.014<br>0.048	1.94 (1.02-3.71)<br>4.17 (1.16-15.08)<br>0.36 (0.16-0.81)<br>0.54 (0.30-0.99)
HCV G1	IFN-λ3/4 rs12979860 CC<br>CA01CA01	0.024<br>0.041	3.07 (1.16-8.14)<br>2.74 (1.04-7.20)
HCV G2/3	KIR2DL3:HLA-C1<br>CB01	0.038<br>0.051	3.33 (1.07-10.39)<br>0.33 (0.11-1.01)
