# Supplementary material for: The interaction of genetic determinants in the outcome of HCV infection: evidence for discrete immunological pathways
Source: Tissue Antigens. 2015 Sep 18;86(4):267–75. doi: 10.1111/tan.12650 (PMC4858811; doi:10.1111/tan.12650)
Supplement: Supplementary file 3 — Table S3. Genetic data available for high resolution KIR typing and treatment related outcomes presented according to haplotype (n = 185). [file TAN-86-267-s003.pdf]

Table 3. Genetic data available for high resolution KIR typing and treatment related outcomes presented according to haplotype (n=185)

	KIR Copy No	All patients n = 185	All SVR n (%)	G1 n = 91	G1 SVR n (%)	G2/3 n = 94	G2/3 SVR n (%)
Haplotype A							
CA01		***155***	***83 (55.7)***	***76***	***28 (37.8)***	***79***	***55 (73.3)***
KIR2DL1	0<br>1<br>2<br>3<br>4	4<br>55<br>112<br>3<br>1	1 (25)<br>28 (50.9)<br>66 (58.9)<br>2 (66.7)<br>1 (100)	2<br>24<br>58<br>1<br>0	1 (50)<br>6 (25)<br>28 (48.3)<br>0 (0)<br>0 (0)	2<br>31<br>54<br>2<br>1	0 (0)<br>22 (71)<br>38 (70.4)<br>2 (100)<br>1 (100)
KIR2DL3	0<br>1<br>2	11<br>85<br>77	6 (54.5)<br>43 (50.6)<br>48 (62.3)	5<br>42<br>37	4 (80)<br>12 (28.6)<br>18 (48.6)	6<br>43<br>40	2 (33.3)<br>31 (72.1)<br>30 (75)
KIR 2DP1	0<br>1<br>2<br>3	3<br>54<br>115<br>3	1 (33.3)<br>27 (50)<br>68 (59.1)<br>2 (66.7)	2<br>23<br>59<br>1	1 (50)<br>6 (26.1)<br>28 (47.5)<br>0 (0)	1<br>31<br>56<br>2	0 (0)<br>21 (67.7)<br>40 (71.4)<br>2 (100)
TA01		***156***	***83 (54.6)***	***76***	***29 (39.2)***	***80***	***54 (69.2)***
KIR 3DL1	0<br>1<br>2<br>3	10<br>58<br>106<br>1	7 (70)<br>35 (60.3)<br>55 (51.9)<br>1 (100)	5<br>24<br>55<br>1	2 (40)<br>12 (50)<br>20 (36.4)<br>1 (100)	5<br>34<br>51<br>0	5 (100)<br>23 (67.6)<br>35 (68.6)<br>0 (0)
KIR 2DS4	0<br>1<br>2	10<br>59<br>106	7 (70)<br>35 (59.3)<br>56 (52.8)	5<br>25<br>55	2 (40)<br>12 (48)<br>21 (38.2)	5<br>34<br>51	5 (100)<br>23 (67.6)<br>35 (68.6)
Haplotype B							
CB01		***147***	***16 (44.4)***	***73***	***7 (38.9)***	***74***	***9 (50)***
KIR2DL2	0<br>1<br>2	83<br>82<br>10	53 (63.9)<br>40 (48.8)<br>5 (50)	38<br>43<br>4	19 (50)<br>13 (30.2)<br>3 (75)	45<br>39<br>6	34 (75.6)<br>27 (69.2)<br>2 (33.3)
KIR 2DL5	0<br>1<br>2<br>3<br>4	89<br>58<br>22<br>5<br>1	49 (55.1)<br>31 (53.4)<br>15 (68.2)<br>2 (40)<br>1 (100)	44<br>29<br>8<br>3<br>1	17 (38.6)<br>11 (37.9)<br>6 (75)<br>0 (0)<br>1 (100)	45<br>29<br>14<br>2<br>0	32 (71.1)<br>20 (69.0)<br>9 (64.3)<br>2 (100)<br>0 (0)
KIR 2DS2	0<br>1<br>2	81<br>85<br>9	51 (63)<br>42 (49.4)<br>5 (55.6)	38<br>43<br>4	19 (50)<br>13 (30.2)<br>3 (75)	43<br>42<br>5	32 (74.4)<br>29 (69)<br>2 (40)
KIR 2DS3	0<br>1<br>2<br>3	128<br>35<br>10<br>2	76 (59.4)<br>13 (37.1)<br>8 (80)<br>1 (50)	59<br>19<br>5<br>2	25 (42.4)<br>5 (26.3)<br>4 (80)<br>1 (50)	69<br>16<br>5<br>0	51 (73.9)<br>8 (50)<br>4 (80)<br>0 (0)
TB01		***150***	***32 (58.2)***	***75***	***10 (41.7)***	***75***	***22 (71)***
KIR 2DS1	0<br>1<br>2	108<br>57<br>10	57 (52.8)<br>34 (59.6)<br>7 (70)	56<br>24<br>5	21 (37.5)<br>12 (50)<br>2 (40)	52<br>33<br>5	36 (69.2)<br>22 (66.7)<br>5 (100)
KIR 2DS5	0	122	64 (52.5)	64	25 (39.1)	58	39 (67.2)

	1<br>2	48<br>5	30 (62.5)<br>4 (80)	19<br>2	9 (47.4)<br>1 (50)	29<br>3	21 (72.4)<br>3 (100)
KIR 3DS1	0<br>1<br>2<br>3	109<br>55<br>8<br>3	56 (51.4)<br>35 (63.6)<br>5 (62.5)<br>2 (66.7)	57<br>23<br>3<br>2	21 (36.8)<br>13 (56.5)<br>0 (0)<br>1 (50)	52<br>32<br>5<br>1	35 (67.3)<br>2 (68.8)<br>5 (100)<br>1 (100)
Framework							
KIR 2DL4	0<br>1<br>2	5<br>161<br>8	2 (40)<br>90 (55.9)<br>5 (62.5)	2<br>78<br>4	1 (50)<br>31 (39.7)<br>2 (50)	3<br>83<br>4	1 (33.3)<br>59 (71.1)<br>3 (75)
KIR 3DL2	1<br>2	7<br>168	3 (42.9)<br>95 (56.5)	2<br>83	0 (0)<br>35 (42.2)	5<br>85	3 (60)<br>60 (70.6)
KIR 3DL3	2	175	98 (56)	85	35 (41.2)	90	63 (70)
KIR 3DP1	1<br>2<br>3	4<br>164<br>7	1 (25)<br>93 (56.7)<br>4 (57.1)	2<br>80<br>3	1 (50)<br>33 (41.3)<br>1 (33.3)	2<br>84<br>4	0 (0)<br>60 (71.4)<br>3 (75)
